# Identification of therapeutic targets and prognostic biomarkers among frizzled family genes in glioma

**DOI:** 10.3389/fmolb.2022.1054614

**Published:** 2023-01-09

**Authors:** Ke Huang, Huimei Xu, Liang Han, Ruiming Xu, Zhaoqing Xu, Yi Xie

**Affiliations:** ^1^ School of Basic Medical Sciences, Lanzhou University, Lanzhou, China; ^2^ School/Hospital of Stomatology, Lanzhou University, Lanzhou, China; ^3^ Lanzhou University Second Hospital, Lanzhou University, Lanzhou, China; ^4^ The Second Hospital of Dalian Medical University, Dalian, China; ^5^ Institute of Modern Physics, Chinese Academy of Sciences, Lanzhou, China; ^6^ Key Laboratory of Heavy Ion Radiation Biology and Medicine of Chinese Academy of Sciences, Lanzhou, China; ^7^ Key Laboratory of Heavy Ion Radiation Medicine of Gansu Province, Lanzhou, China

**Keywords:** frizzled gene family, MTOR signaling, biomarker, therapeutic targets, glioma

## Abstract

**Background:** The biological functions of the Frizzled gene family (FZDs), as the key node of wingless-type MMTV integration site family (Wnt) and mammalian target of rapamycin signaling pathways, have not been fully elucidated in glioma. This study aims to identify novel therapeutic targets and prognostic biomarkers for gliomas, which may help us understand the role of FZDs.

**Methods:** RNA-sequence data were downloaded from The Cancer Genome Atlas (TCGA) and Genotype-Tissue Expression (GTEx) projects. Survival analyses, Cox regression analyses, nomograms, calibration curves, receiver operating characteristic (ROC) curves, gene function enrichment analyses, and immune cell infiltration analyses were conducted using R.

**Results:** High expressions of FZDs were positively associated with the activation of mTOR signaling. FZD1/2/3/4/5/7/8 was significantly highly expressed in tumor tissues, and the high expression of FZD1/2/5/6/7/8 was significantly positively associated with poorer prognosis. FZD2 and FZD6 positively served as independent predictors of poor prognosis. Gene function analysis showed that FZDs were associated with mTOR signaling, immune response, cytokine-cytokine receptor interaction, extracellular matrix organization, apoptosis, and p53 signaling pathway.

**Conclusions:** Our finding strongly indicated a crucial role of FZDs in glioma. FZD1/2/5/6/7/8 could be an unfavorable prognostic factor in glioma and FZD2 and FZD6 may be novel independent predictors of poor prognosis in glioma.

## Highlights

• FZD1/2/5/6/7/8 is an unfavorable prognostic factor for glioma.
• FZD2/6 serves as a novel independent predictor of poor prognosis in glioma.
• FZD2/6 expression is positively correlated with immune checkpoints.
• FZD2/6 is closely connected with immune cell infiltration.

## 1 Introduction

Glioma is one of the most common cancers of the central nervous system. The clinical prognosis for patients with glioma remains chronically poor despite recent advances in understanding the molecular basis of oncogenesis, as well as enhanced neuroimaging technology, surgery, and adjuvant therapy. The best treatment options for gliomas include surgical resection, radiation, and concurrent or adjuvant chemotherapy with the alkylating agent temozolomide (Stupp et al., 2009; Bureta et al., 2019; Garnier et al., 2019). The median survival time of glioma patients is 14–18 months, with few cases of extended overall survival (OS) times reaching 20.9 months (Ho et al., 2014; Garnier et al., 2019; Bozzato et al., 2020). Besides, only less than 5% of glioma patients reach a 5-year survival (Ho et al., 2014; Garnier et al., 2019). Recently, potential therapeutic targets for glioma have been extensively studied (Le Rhun et al., 2019; Tan et al., 2020). Non-etheless, the identification of novel and effective biomarkers that indicate the therapeutic efficacy for glioma is urgent.

Aberrant signaling pathways and regulation of growth factors in glioma cells have been reported. Different signaling pathways play a role in glioma metastasis, invasion, and cellular proliferation. Members of the frizzled gene family (FZDs) encode proteins with seven transmembrane domains that act as receptors for the family of signaling proteins called the wingless-type MMTV integration site family (Wnt). The relationship between the FZD gene family and the Wnt signaling pathway has been widely explored. FZD genes have been broadly considered to participate in cardiovascular development and angiogenesis (Hlubek et al., 2007; van de Schans et al., 2008; Zerlin et al., 2008; MacDonald and He, 2012; Dijksterhuis et al., 2014). Increasing evidence has highlighted the deregulation of the Wnt pathway in several tumors, including glioma. The intensity of the Wnt/β-catenin signaling pathway is incredibly sensitive to FZD protein concentration on the plasma membrane, as it is the primary receptor of Wnt proteins (Hlubek et al., 2007; Shi et al., 2007; Ueno et al., 2013; Shahcheraghi et al., 2020; van Loon et al., 2021). However, despite the critical role of FZDs in Wnt signaling, the specific FZDs and how they are controlled in glioma are yet to be elucidated.

Numerous studies have demonstrated the immunosuppressive feature of the tumor microenvironment (TME) in glioma (Coniglio et al., 2012; Ma et al., 2018; Wei et al., 2019; Xu et al., 2020). It has been proven that changes in tumor-adjacent stroma play a crucial role in tumor progression. Cancer cells can functionally shape their microenvironment through the production of different cytokines, chemokines, and other substances. This leads to the reprogramming of the surrounding cells, which eventually contributes to tumor growth (Hinshaw and Shevde, 2019). Immune cell infiltration varies the tumor stage and is an essential component of the tumor stroma (Bindea et al., 2013). Accumulating data has shown that the presence of innate immune cells in the TME, including macrophages, neutrophils, dendritic cells (DCs), innate lymphoid cells, myeloid-derived suppressor cells, and natural killer (NK) cells, contributes to tumor growth (Roma-Rodrigues et al., 2019). Moreover, immune checkpoint blockade is a promising approach for glioma immunotherapy (Wainwright et al., 2014). Wnt signaling has been reported to induce a proinflammatory signature in glioma (Dijksterhuis et al., 2015). However, the association between FZDs and the TME remains elusive.

Herein, datasets from The Cancer Genome Atlas (TCGA) Genotype-Tissue Expression (GTEx) projects were applied to evaluate the expression of FZDs in glioma, and clinical characteristics and prognostic features related to FZDs were identified. Next, the roles of the genes in the co-expression network were assessed using Gene ontology (GO) and the Kyoto encyclopedia of genes and genomes (KEGG). Moreover, the interrelationships between FZD expression and immune response were explored by evaluating immune cell infiltration and co-expression of immune checkpoint genes and macrophage polarization. Collectively, our finding strongly demonstrated a crucial role of the FZDs in glioma and proved that FZD2 and FZD6 may be novel independent predictors of poor prognosis in glioma.

## 2 Materials and methods

### 2.1 Data collection and integration

The associated clinical and RNA-sequencing data were retrieved from TCGA (Blum et al., 2018), and UCSC Xena (https://xenabrowser.net/datapages/) provided RNA-sequencing data standardized by the Toil process (TCGA and GTEx) (Vivian et al., 2017). A total of 1846 samples were utilized, including 1,152 normal samples from GTEx and 5 tumor surrounding tissues and 689 tumor tissues from TCGA. Afterward, log2 fold change (log 2 FC) was calculated, which was used to compare the messenger RNA (mRNA) expression level between tumor and non-tumor samples. Patient clinical data included age, gender, World Health Organization (WHO) grade, isocitrate dehydrogenase (IDH) status, 1p/19q status, histological type, and OS event. Samples with ambiguous or inaccurate data were excluded.

### 2.2 Survival and statistical analyses

Patients were divided into groups with high and low FZD expression based on the median level of FZD expression. Using the R packages survminer (version 0.4.9) and survival (version 3.2.10), Kaplan-Meier (KM) survival analysis was performed to determine the relationship between the FZD expression level and OS. The survival package was used for the proportional hazards assumption and survival regression. The results were visualized using the survminer package.

### 2.3 Univariate and multivariate cox regression analyses

Univariate and multivariate Cox regression analyses were utilized to determine if clinicopathological features such as FZD expression, age, WHO grade, IDH status, 1p/19q status, and histological type were independent predictive markers of survival in patients with glioma. Variables with a *p*-value <0.1 in the univariate analysis were included in the multivariate Cox model to identify independent prognostic factors. Hazard ratios (HR) and 95% confidence intervals (CI) were computed, and a significance level of *p* < 0.05 was used. The survival package in R (version 3.2.10) was utilized for data analysis.

### 2.4 Construction of nomograms, calibration curves, and receiver operating characteristic (ROC) curves

A nomogram was employed to forecast the prognosis of cancer using R packages survival (version 3.2.10) and rms (version 6.3.0). The survival package was used for the proportional hazards assumption and Cox regression analysis, and the rms package was used for the construction and visualization of the nomogram model. Calibration curves were drawn to evaluate the deviation of estimated probabilities from ideal values. Parameters for the calibration analysis were set as follows: the number of samples in each group of repeated calculations (40); the number of double counts (200); method (boot). Using the R packages pROC (version 1.17.0.1) and ggplot2 (version 3.3.3), time-dependent ROC curves were produced for diagnostic analysis.

### 2.5 FZDs-related function enrichment analyses

Differentially expressed genes (DEGs) were analyzed using the R package DESeq2 (version 1.26.0) (Love et al., 2014). Gene ontology (GO) and Kyoto encyclopedia of genes and genomes (KEGG) analyses were performed to assess putative gene functions related to FZDs based on the TCGA database using R packages org. Hs.eg.db (version 3.10.0) and clusterProfiler (version 3.14.3) (Yu et al., 2012). The Gene Set Enrichment Analysis (GSEA) computational method—which assesses the statistical significance of a predetermined set of genes and the existence of concordant differences between two biological states—was performed using the R package clusterProfiler (version 3.14.3) (Subramanian et al., 2007) and the results were visualized using the R package ggplot2 (version 3.3.3) was also used for data visualization. Gene sets were assessed in GSEA analyses using the adjusted *p*-value (p.adj), false discovery rate (FDR), and the absolute value of the normalized enrichment score (NES). Significant enrichment was defined as |NES|>1, p. adj<0.05, and FDR <0.25 for gene sets.

### 2.6 Immune cell infiltration analyses

The relationship between FZD expression and the infiltrating immune cells was examined using the R package GSVA (version 1.34.0) (Hänzelmann et al., 2013). Twenty-four different types of immune cells in gliomas were explored (Bindea et al., 2013).

### 2.7 Statistical analyses

R software (version 3.6.3) was used for statistical analysis and graphing. The R package used in this study included survminer, survival, rms, pROC, ggplot2, DESeq2, org. Hs.eg.db, clusterProfiler, and GSVA. The Adobe Photoshop software (version 21.0.1) was used for image editing and design. Cox regression analysis was used to determine the correlation between clinical data and gene expression. *p* < 0.05 served as the cut-off value.

## 3 Results

### 3.1 Clinical characteristics of the glioma patients

The TCGA database provided the clinical and gene expression data for 698 primary tumors and 5 tumor surrounding tissues, whereas the GTEx database provided the data for 1,152 normal samples. Patient clinical data included age, WHO classification, IDH status, 1q/19p codeletion, and OS event. Supplementary data of the WHO grade, IDH status, and 1q/19p codeletion were from a study by Ceccarelli et al. (2016). Samples with ambiguous or inaccurate data were discarded (Tables 1–4). The results showed that high expression of FZD1/2/5/6/7/8/10 was positively correlated with the dead group (OS event), and FZD9 was correlated with the alive group (OS event). Meanwhile, FZD3 and FZD4 showed no clear correlation between clinical features and gene expression.

**TABLE 1:** The FZDs expressions in glioma patients with different clinical parameters.

Characteristic	FZD1			FZD2			FZD3		
	Low	High	*p*	Low	High	*p*	Low	High	*p*
N	348	348		348	348		348	348	
Age, meidan (IQR)	40 (32, 51.25)	52.5 (38, 62)	<0.001	40 (32, 52	52 (38, 63)	<0.001	46.5 (35, 59)	45 (33, 58)	0.190
Age, n (%)	—	—	<0.001	—	—	<0.001	—	—	0.260
<=60	308 (44.3%)	245 (35.2%)	—	310 (44.5%)	243 (34.9%)	—	270 (38.8%)	283 (40.7%)	—
>60	40 (5.7%)	103 (14.8%)	—	38 (5.5%)	105 (15.1%)	—	78 (11.2%)	65 (9.3%)	—
WHO grade, n (%)	—	—	<0.001	—	—	<0.001	—	—	0.902
G2	153 (24.1%)	71 (11.2%)	—	178 (28%)	46 (7.2%)	—	112 (17.6%)	112 (17.6%)	—
G3	131 (20.6%)	112 (17.6%)	—	120 (18.9%)	123 (19.4%)	—	117 (18.4%)	126 (19.8%)	—
G4	20 (3.1%)	148 (23.3%)	—	17 (2.7%)	151 (23.8%)	—	84 (13.2%)	84 (13.2%)	—
IDH status, n (%)	—	—	<0.001	—	—	<0.001	—	—	0.042
WT	31 (4.5%)	215 (31.3%)	—	39 (5.7%)	207 (30.2%)	—	109 (15.9%)	137 (20%)	—
Mut	312 (45.5%)	128 (18.7%)	—	303 (44.2%)	137 (20%)	—	232 (33.8%)	208 (30.3%)	—
1p/19q codeletion, *n* (%)	—	—	<0.001	—	—	<0.001	—	—	0.032
Codel	135 (19.6%)	36 (5.2%)		138 (20%)	33 (4.8%)		99 (14.4%)	72 (10.4%)	
non-codel	212 (30.8%)	306 (44.4%)		210 (30.5%)	308 (44.7%)		249 (36.1%)	269 (39%)	
OS event, n (%)	—	—	<0.001	—	—	<0.001	—	—	0.393
Alive	267 (38.4%)	157 (22.6%)	—	284 (40.8%)	140 (20.1%)	—	218 (31.3%)	206 (29.6%)	—
Dead	81 (11.6%)	191 (27.4%)	—	64 (9.2%)	208 (29.9%)	—	130 (18.7%)	142 (20.4%)	—

**TABLE 2:** The FZDs expressions in glioma patients with different clinical parameters.

Characteristic	FZD4			FZD5			FZD6		
	Low	High	*p*	Low	High	*p*	Low	High	*p*
N	348	348	—	348	348	—	348	348	—
Age, meidan (IQR)	44 (35, 58)	46.5 (33, 59)	0.647	41 (33.75, 53.25)	51 (35.75, 62)	<0.001	39 (32, 51.25)	53 (39, 62)	<0.001
Age, n (%)	—	—	0.260	—	—	<0.001	—	—	<0.001
<=60	283 (40.7%)	270 (38.8%)	—	302 (43.4%)	251 (36.1%)	—	306 (44%)	247 (35.5%)	—
>60	65 (9.3%)	78 (11.2%)	—	46 (6.6%)	97 (13.9%)	—	42 (6%)	101 (14.5%)	—
WHO grade, n (%)	—	—	<0.001	—	—	<0.001	—	—	<0.001
G2	132 (20.8%)	92 (14.5%)	—	153 (24.1%)	71 (11.2%)	—	168 (26.5%)	56 (8.8%)	—
G3	100 (15.7%)	143 (22.5%)	—	133 (20.9%)	110 (17.3%)	—	112 (17.6%)	131 (20.6%)	—
G4	81 (12.8%)	87 (13.7%)	—	24 (3.8%)	144 (22.7%)	—	21 (3.3%)	147 (23.1%)	—
IDH status, n (%)	—	—	0.004	—	—	<0.001	—	—	<0.001
WT	103 (15%)	143 (20.8%)	—	53 (7.7%)	193 (28.1%)		31 (4.5%)	215 (31.3%)	—
Mut	236 (34.4%)	204 (29.7%)	—	291 (42.4%)	149 (21.7%)		314 (45.8%)	126 (18.4%)	—
1p/19q codeletion, n (%)	—	—	<0.001	—	—	<0.001	—		<0.001
Codel	65 (9.4%)	106 (15.4%)	—	134 (19.4%)	37 (5.4%)	—	117 (17%)	54 (7.8%)	—
non-codel	279 (40.5%)	239 (34.7%)	—	214 (31.1%)	304 (44.1%)	—	228 (33.1%)	290 (42.1%)	—
OS event, n (%)	—	—	0.698	—	—	<0.001	—	—	<0.001
Alive	215 (30.9%)	209 (30%)	—	267 (38.4%)	157 (22.6%)	—	278 (39.9%)	146 (21%)	—
Dead	133 (19.1%)	139 (20%)	—	81 (11.6%)	191 (27.4%)	—	70 (10.1%)	202 (29%)	—

**TABLE 3:** The FZDs expressions in glioma patients with different clinical parameters.

Characteristic	FZD7			FZD8		
	Low	High	*p*	Low	High	*p*
N	348	348		348	348	
Age, meidan (IQR)	42 (33, 54)	49 (36, 62)	<0.001	43 (34, 55.25)	48 (35, 61)	0.002
Age, n (%)	—	—	<0.001	—	—	<0.001
≤60	296 (42.5%)	257 (36.9%)	—	295 (42.4%)	258 (37.1%)	—
>60	52 (7.5%)	91 (13.1%)	—	53 (7.6%)	90 (12.9%)	—
WHO grade, n (%)	—	—	<0.001	—	—	<0.001
G2	136 (21.4%)	88 (13.9%)	—	125 (19.7%)	99 (15.6%)	—
G3	144 (22.7%)	99 (15.6%)	—	133 (20.9%)	110 (17.3%)	—
G4	31 (4.9%)	137 (21.6%)	—	52 (8.2%)	116 (18.3%)	—
IDH status, n (%)	—	—	<0.001	—	—	<0.001
WT	58 (8.5%)	188 (27.4%)	—	72 (10.5%)	174 (25.4%)	
Mut	287 (41.8%)	153 (22.3%)	—	270 (39.4%)	170 (24.8%)	—
1p/19q codeletion, *n* (%)	—	—	<0.001	—	—	<0.001
Codel	130 (18.9%)	41 (6%)	—	112 (16.3%)	59 (8.6%)	—
non-codel	216 (31.3%)	302 (43.8%)		233 (33.8%)	285 (41.4%)	—
OS event, n (%)	—	—	<0.001	—	—	0.004
Alive	252 (36.2%)	172 (24.7%)	—	231 (33.2%)	193 (27.7%)	—
Dead	96 (13.8%)	176 (25.3%)	—	117 (16.8%)	155 (22.3%)	—

**TABLE 4:** The FZDs expressions in glioma patients with different clinical parameters.

Characteristic	FZD9			FZD10		
	Low	High	*p*	Low	High	*p*
N	348	348	—	348	348	—
Age, meidan (IQR)	51 (38, 62)	40 (31, 54)	<0.001	42 (33, 54.25)	49.5 (36, 61)	<0.001
Age, n (%)	—	—	<0.001	—	—	<0.001
≤60	252 (36.2%)	301 (43.2%)	—	297 (42.7%)	256 (36.8%)	—
>60	96 (13.8%)	47 (6.8%)	—	51 (7.3%)	92 (13.2%)	—
WHO grade, n (%)	—	—	<0.001	—	—	<0.001
G2	87 (13.7%)	137 (21.6%)	—	137 (21.6%)	87 (13.7%)	—
G3	112 (17.6%)	131 (20.6%)	—	104 (16.4%)	139 (21.9%)	—
G4	120 (18.9%)	48 (7.6%)	—	65 (10.2%)	103 (16.2%)	—
IDH status, n (%)	—	—	<0.001	—	—	<0.001
WT	148 (21.6%)	98 (14.3%)	—	89 (13%)	157 (22.9%)	—
Mut	194 (28.3%)	246 (35.9%)	—	253 (36.9%)	187 (27.3%)	—
1p/19q codeletion, n (%)	—	—	0.002	—	—	0.152
Codel	103 (14.9%)	68 (9.9%)	—	77 (11.2%)	94 (13.6%)	—
non-codel	239 (34.7%)	279 (40.5%)	—	268 (38.9%)	250 (36.3%)	—
OS event, n (%)	—	—	0.007	—	—	0.007
Alive	194 (27.9%)	230 (33%)	—	230 (33%)	194 (27.9%)	—
Dead	154 (22.1%)	118 (17%)	—	118 (17%)	154 (22.1%)	—

### 3.2 Gene expressions of FZDs in glioma

**FIGURE 1:**
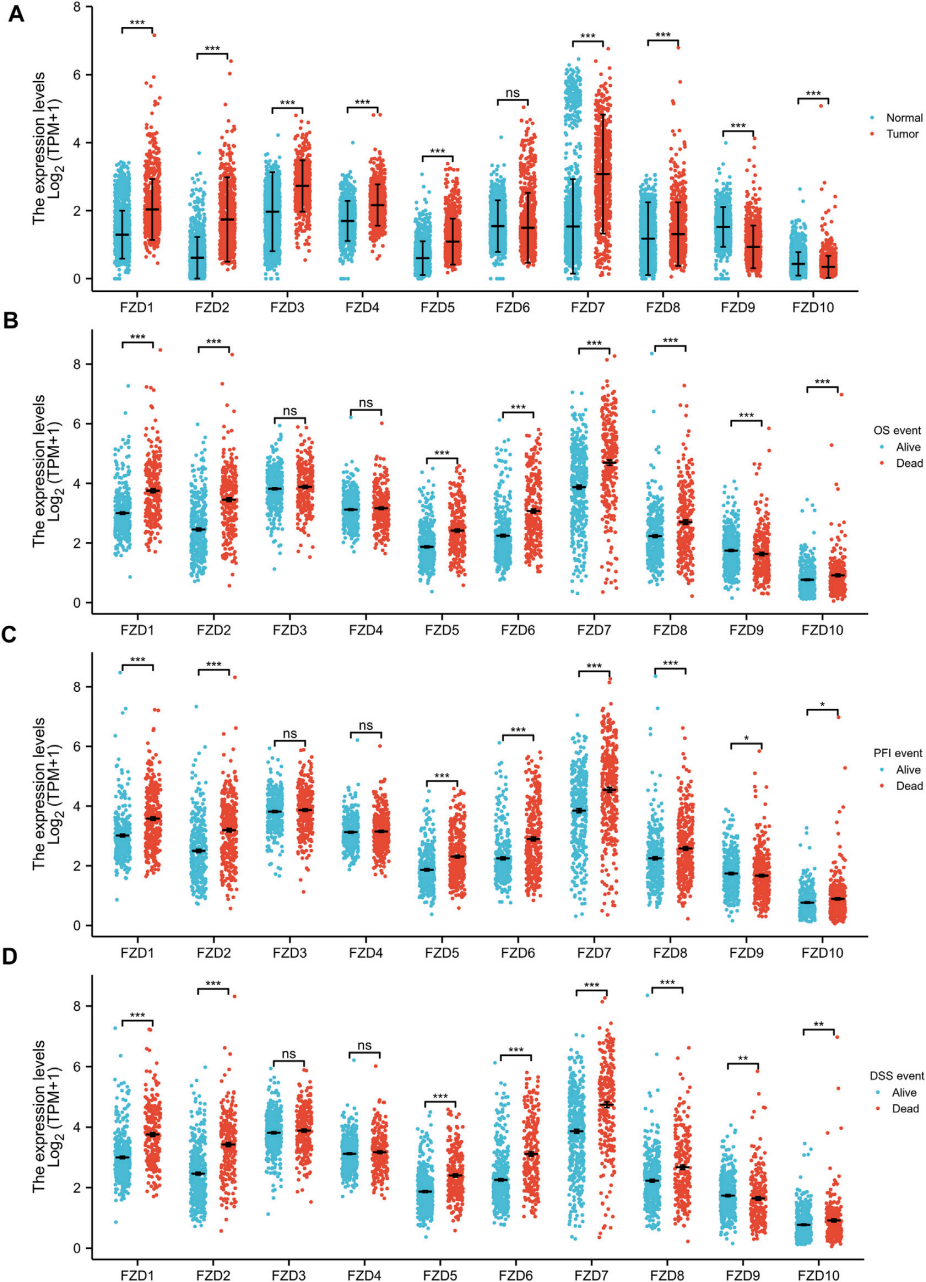
Wilcoxon rank sum test of differentially expressed genes (DEGs). **(A)** Gene expression levels of FZDs in normal tissues and gliomas. **(B–D)** Correlation between FZD expression and the overall survival event (OS event), progression-free interval event (PFI event), and disease-free survival event (DSS event).

**FIGURE 2:**
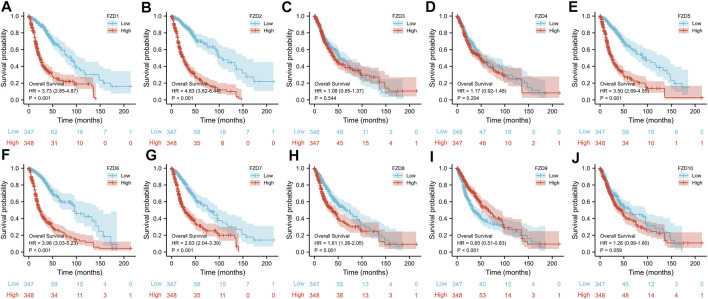
Kaplan-Meier (KM) curves of the association between FZD expression and overall survival. **(A–J)** KM curves of FZD1-10.

FZD1/2/3/4/5/7/8 expression was significantly higher in tumor tissues than in normal brain tissues; however, FZD9 and FZD10 expression was higher in normal tissues (Figure 1A). Next, the correlations between the expressions of FZDs and different prognostic groups were explored. Results showed that FZD1/2/5/6/7/8/10 was more highly expressed in dead groups, while FZD9 was highly expressed in alive groups (Figures 1B–D). KM survival analysis was performed to determine the relationship between FZD expressions and OS of glioma patients. Patients were categorized into groups with high and low gene expression based on the median expression level of FZDs. The KM curves revealed that a high expression level of FZD1/2/5/6/7/8 was positively correlated with poor OS (Figure 2).

### 3.3 Correlations between clinical characteristics and the expressions of FZDs in glioma

**FIGURE 3:**
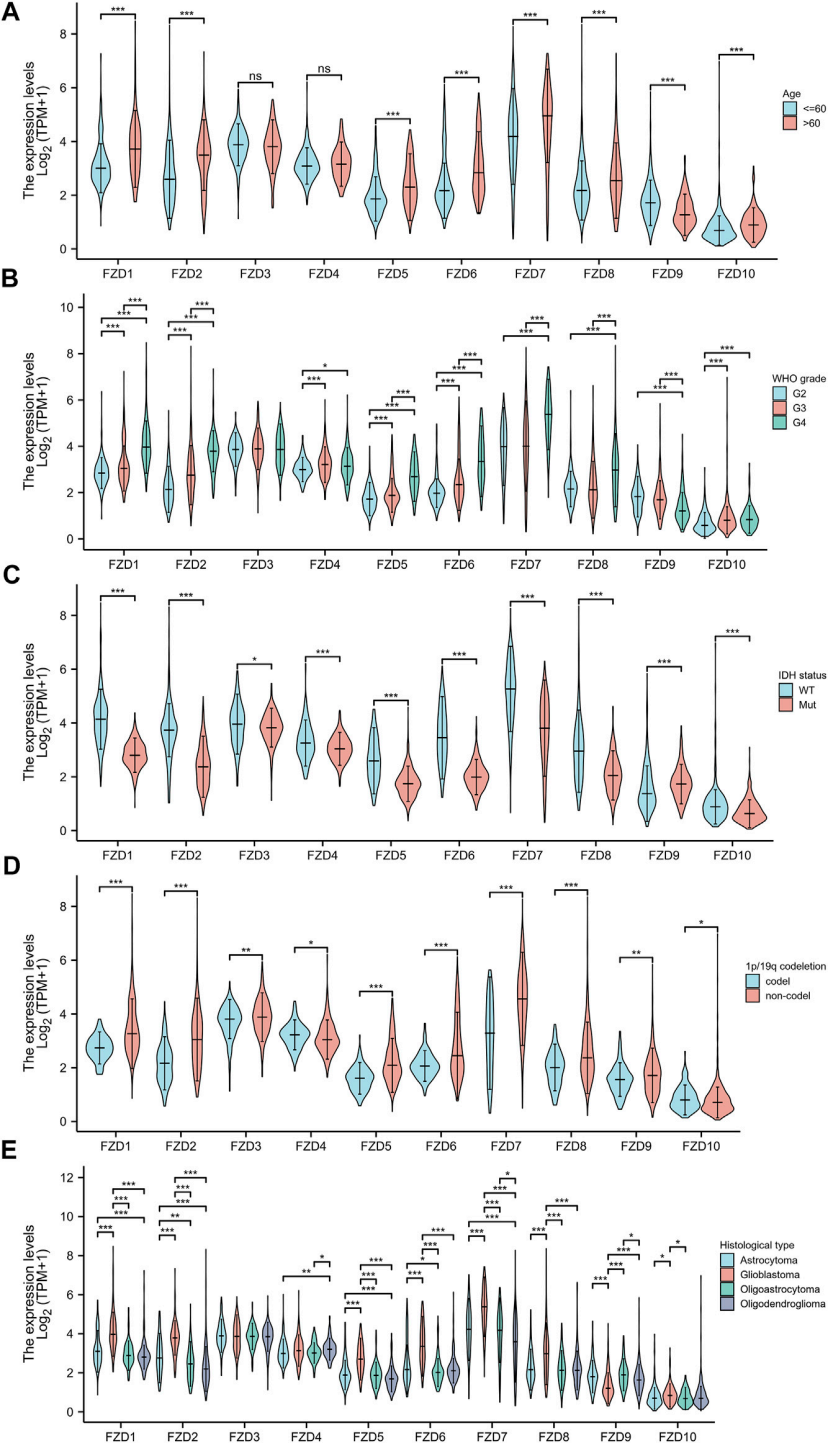
Association between FZD expression and clinical characteristics. **(A)** Age. **(B)** WHO grade. **(C)** IDH status. **(D)** 1p/19q status. **(E)** Histological type.

The Kruskal–Wallis test was used to examine the correlations between clinical traits and FZD mRNA expressions in gliomas. Results showed that the high expression level of FZD1/2/5/6/7/8/10 was positively associated with the high age group (Figure 3A). A marked increase in the expression of FZD1/2/4/5/6/7/8/10 was positively associated with the progression of gliomas from grade II to grade IV. However, decreased expression of FZD9 was positively associated with the progression of gliomas from grade II to grade IV (Figure 3B). Mutations in IDH have been reported in various cancers (Yang et al., 2012). IDH mutations in gliomas were first shown to be more prevalent in lower-grade gliomas and secondary glioblastoma multiformes (GBMs) than in main GBMs (Yan et al., 2009) and were often thought to be connected to improved prognoses (Wick et al., 2009; Dunn et al., 2013; Suzuki et al., 2015). Moreover, a lower expression level of FZD1/2/3/4/5/6/7/8/10 was positively correlated with IDH mutation, suggesting that a high expression of FZD1/2/3/4/5/6/7/8/10 might be associated with a worse prognostic outcome (Figure 3C). Since 1998, the co-deletion of chromosomes 1p and 19q (codel group) has been considered a diagnostic and prognostic sign (Cairncross et al., 1998). Elevated expression of FZD1/2/3/5/6/7/8/9 was observed in the non-codel group (Figure 3D). Furthermore, it was observed that the GBM group had the highest expression level of FZD1/2/5/6/7/8/10 (Figure 3E).

### 3.4 Diagnostic value of FZDs in glioma

**FIGURE 4:**
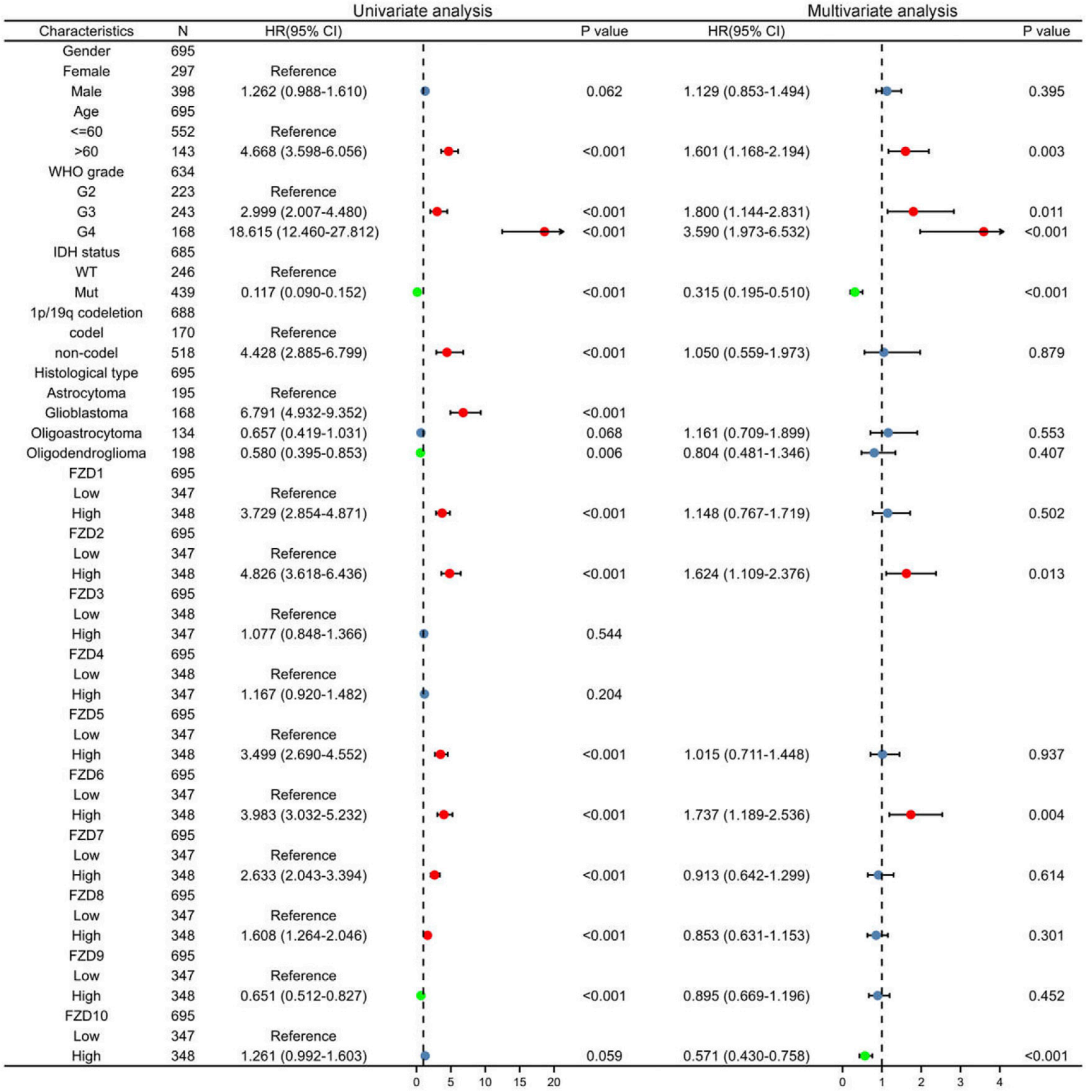
Univariate and multivariate Cox regression analysis. Red dots indicate disadvantageous factors (HR > 1 and *p* < 0.05); green dots indicate protective factors (HR < 1 and *p* < 0.05).

**FIGURE 5:**
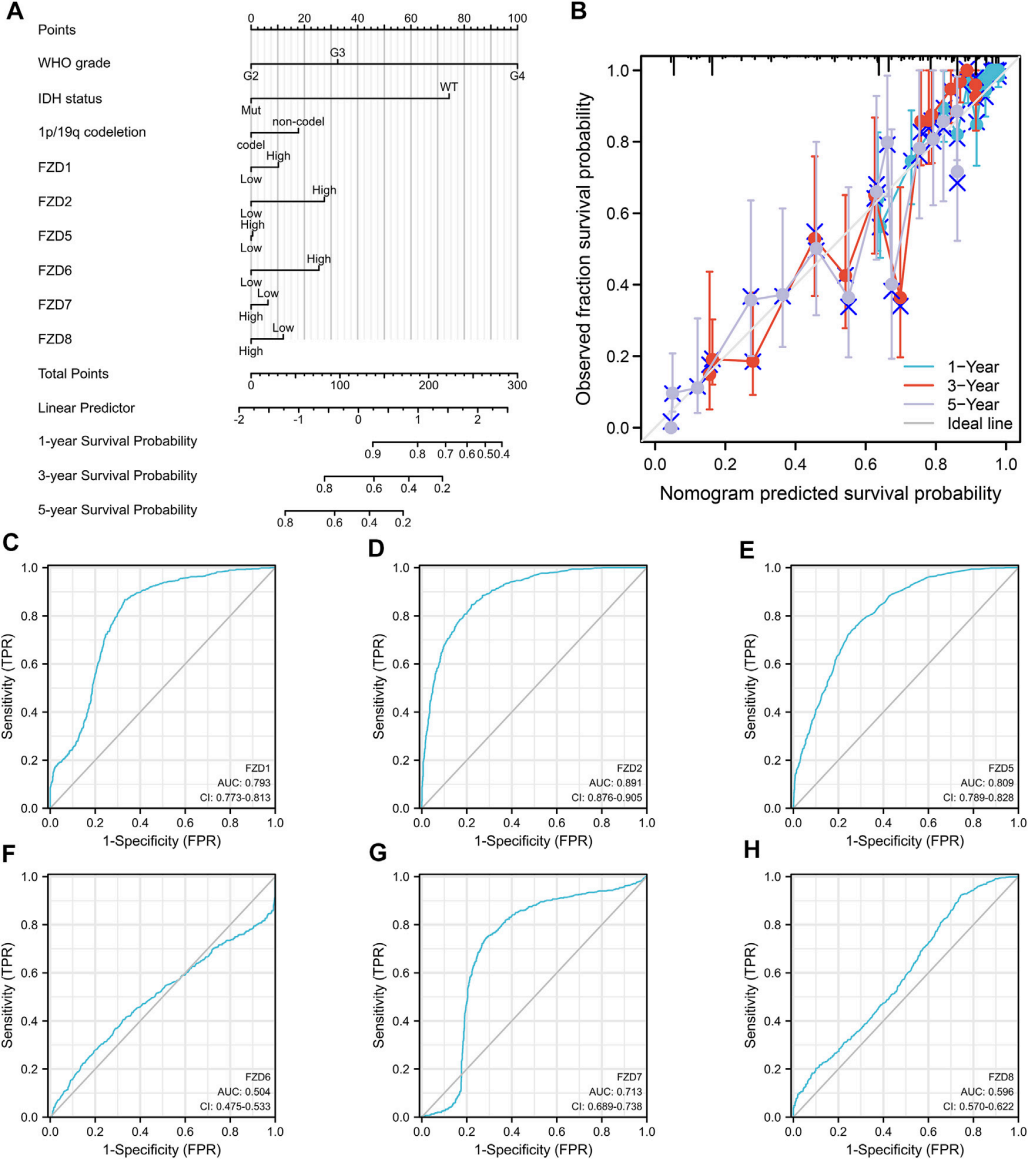
Diagnostic value of FZD1/2/5/6/7/8 in glioma. **(A)** Construction of the nomogram by integrating the expression of FZD1/2/5/6/7/8 with key clinical characteristics. **(B)** Calibration curve of the nomogram for predicting the overall survival at 1, 3, and 5 years. **(C–H)** Diagnostic value of FZD1, FZD2, and FZD5-8 mRNA expression.

Univariate and multivariate Cox regression analyses were used to assess independent risk factors. Multivariate analysis showed that high expression of FZD2 or FZD6 was an independent prognostic factor for poor prognosis. Meanwhile, univariate analysis revealed that high expression of FZD1/2/5/6/7/8 was an unfavorable factor in glioma (Figure 4). Since FZD1/2/5/6/7/8 exhibited characteristics associated with poor prognosis, a nomogram was established with FZD1/2/5/6/7/8 to predict the 1-, 3-, and 5-year survival probability (Figure 5A). A calibration curve was used to evaluate the prediction effect of the nomogram model (WHO grade, IDH status, 1p/19q status, and FZD1/2/5-8 taken as a whole). The ordinate represents the observed survival probability, the gray diagonal represents the ideal line, and the abscissa represents the survival probability predicted by the nomogram model. The lines and points of different colors (except the gray diagonal) represent the situation at different time points predicted by the model. The closer the lines of different colors are to the line of the gray ideal situation, the smaller the error line is (which means the prediction results are stable) (Figure 5B). The established nomogram displayed a superior prediction power. In addition, the diagnostic value of FZD1/2/5/6/7/8 mRNA expression was evaluated by ROC curves. The area under the curve (AUC) of FZD1/2/5/7 suggested an excellent diagnostic value (AUC >0.7), while the AUC of FZD6 and FZD8 only showed an acceptable diagnostic value (0.5 < AUC <0.7) (Figures 5C–H). Taken together, these findings suggest that FZD2 and FZD6 may serve as independent predictors of poor prognosis in glioma, and FZD1/5/7/8 may be a disadvantageous factor.

### 3.5 Predicted gene functions of FZD2 and FZD6

**FIGURE 6:**
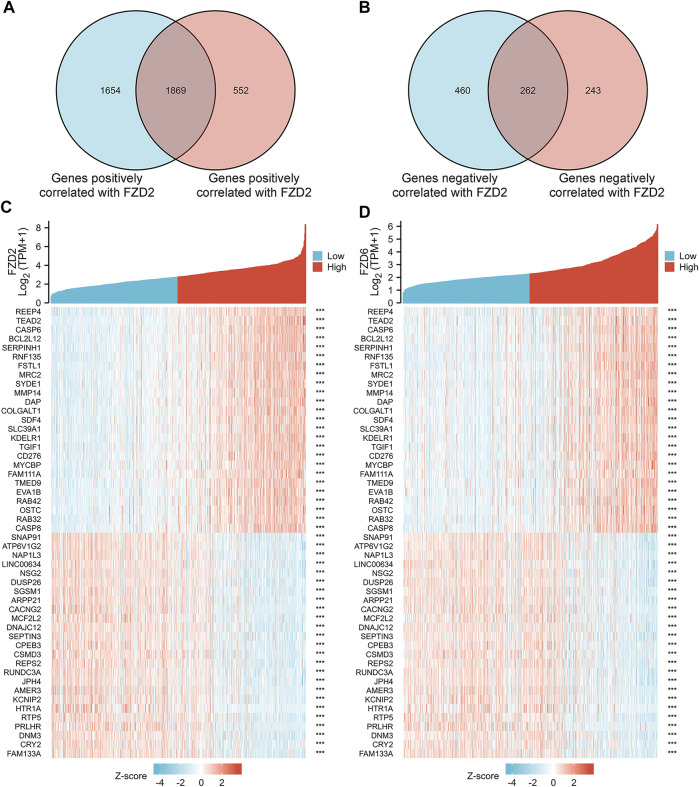
**(A,B)** Gene correlation analysis of FZD2 and FZD6 in Venn diagrams. **(C,D)** The top 25 genes positively or negatively correlated with FZD2 and FZD6 respectively.

**FIGURE 7:**
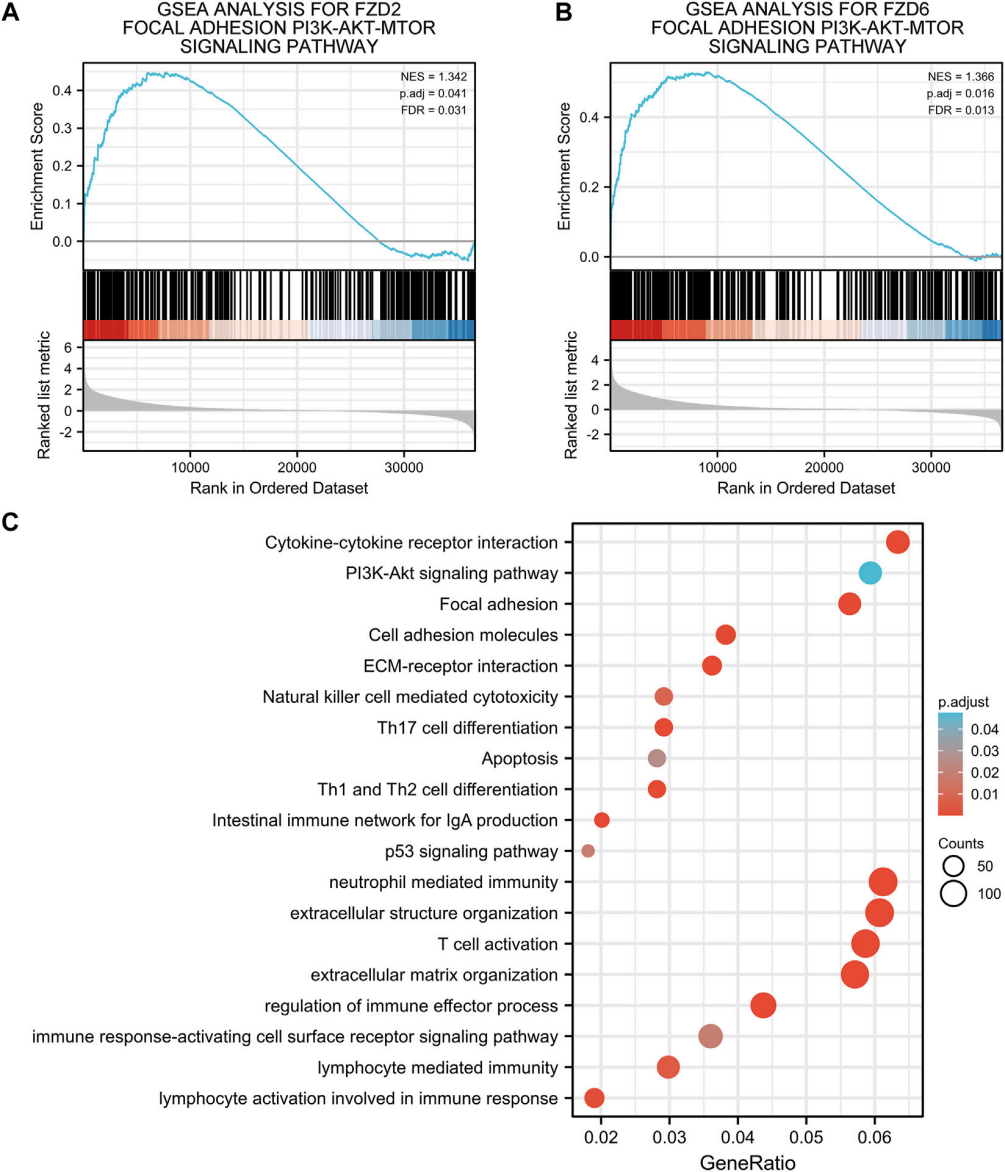
**(A,B)** Gene set enrichment analysis of FZD2 and FZD6. **(C)** Gene ontology and Kyoto encyclopedia of genes and genomes analyses of genes correlated with both FZD2 and FZD6.

Gene correlation analysis was carried out to further understand the gene functions of FZD2 and FZD6. Results showed that 3,523 genes and 2,421 genes were positively correlated with FZD2 and FZD6, respectively. Meanwhile, 722 genes and 505 genes were negatively correlated with FZD2 and FZD6, respectively. Genes correlated with both FZD2 and FZD6 were grouped and organized in Venn diagrams (Figures 6A, B). The top 25 genes positively or negatively correlated with both FZD2 and FZD6 were displayed in heatmaps (Figures 6C, D). Next, GO and KEGG analyses were carried out to assess the functional enrichments of genes correlated with both FZD2 and FZD6. The results showed that FZD2 and FZD6 expressions were related to cytokine-cytokine receptor interaction, extracellular matrix organization, apoptosis, p53 signaling pathway, and immune response (Figure 7C). Furthermore, GSEA analysis was carried out for FZD2 and FZD6 gene function prediction and the results revealed a link between high levels of FZD2 or FZD6 expression and the focal adhesion PI3K-Akt-mTOR signaling pathway (Figures 7A, B).

### 3.6 Correlation between FZD2/6 expression and immune response in glioma

**FIGURE 8:**
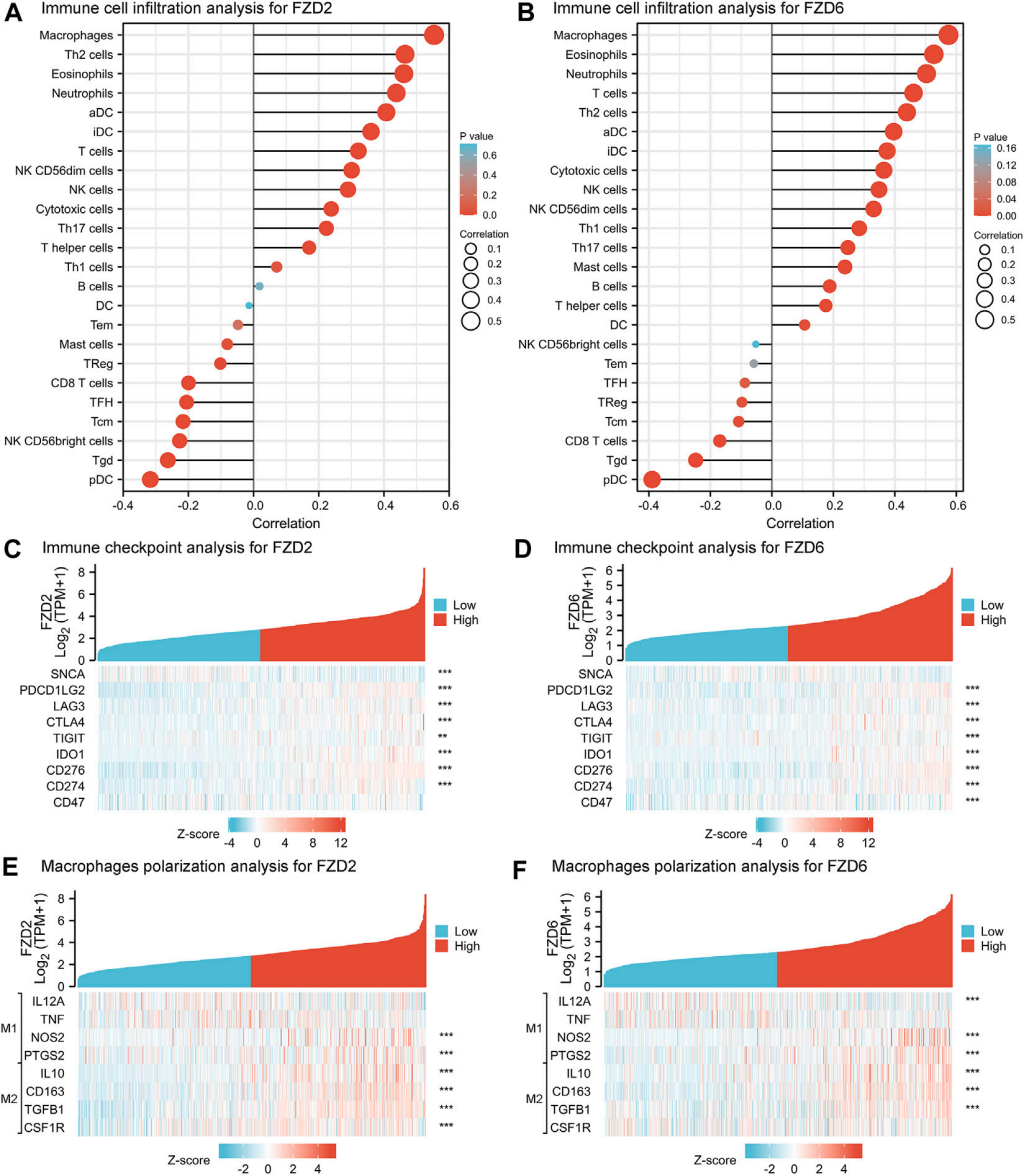
**(A,B)** Immune cell infiltration analysis of FZD2 and FZD6. **(C,D)** Immune checkpoint analysis of FZD2 and FZD6. **(E,F)** Macrophages polarization analysis of FZD2 and FZD6.

Given that there was a significant correlation between immune response and the expression of FZD2 and FZD6, immune cell infiltration was investigated. Infiltrations of macrophages, T helper 2 (Th2) cells, eosinophils, neutrophils, activated DC (aDCs), immature DC (iDCs), T-cell, NK CD56dim cells, NK cells, cytotoxic cells, Th17 cells, T helper cells, and Th1 cells were all positively associated with the expression of FZD2 and FZD6 (Figures 8A, B). To further verify the correlations between FZD2/6 expression and immune response in glioma, we explored the co-expression relationships between FZD2/6 and the key genes related to immune checkpoints (Zhang, D et al., 2021; Yang et al., 2022). The results revealed that FZD2 and FZD6 expressions were positively associated with most of the key genes associated with immune checkpoints (Figures 8C,D). Next, we investigated the essential genes associated with macrophage polarization. It was discovered that expressions of FZD2 and FZD6 (especially FZD2) were strongly and positively correlated with M2-type markers of tumor-associated macrophages (TAMs) (Figures 8E,F). Overall, the correlation between FZD2/6 expression and immune response in glioma was confirmed.

## 4 Discussion

Glioma is the most commonly diagnosed primary tumor of the central nervous system and one of the most invasive malignant tumors in humans. The prognosis of glioma remains poor owing to its extremely invasive and infiltrative properties. Therefore, the identification of reliable predictive biomarkers that indicate the therapeutic efficacy for glioma is urgent.

The mTOR kinase is implicated in the regulation of protein synthesis, cell growth, and cell survival. It is often believed that the PI3K/Akt pathway controls how mTOR responds to growth factor signals. In the present study, GSEA analysis revealed a positive association between high expression levels of FZD2 or FZD6 with the focal adhesion PI3K-Akt-mTOR signaling pathway. Considering the role of FZDs in mTOR signaling, we investigated the expression and the related prognostic value of FZDs. It was found that FZD1/2/3/4/5/7/8 was significantly highly expressed in tumor tissues, and the high expression of FZD1/2/5/6/7/8 was significantly positively associated with a poorer prognosis. This results corroborated with several recent studies. Tompa’s team found that FZD2 was highly expressed in all GBM subgroups (Tompa et al., 2019). Ádám et al. found that FZD2 expression was positively correlated with the glioma grade (Ádám et al., 2021). Zhang et al. demonstrated that FZD6 is a direct target of miR-935, and the expression of FZD6 has a strong correlation with tumor malignancy and prognosis in glioma (Zhang, H et al., 2021). Moreover, our findings confirmed that the high expression of FZD1/2/5/6/7/8 was a disadvantageous factor in glioma, while FZD2 and FZD6 could positively serve as independent predictors of poor prognosis.

The TME has been proven to participate in glioma development. Active communication between tumor cells, adjacent healthy cells, and the surrounding immunological environment encourages the development of cancer and increases resistance to treatment (Barthel et al., 2022). Given that a close connection was found between FZD expression and immune response, we investigated immune infiltration, immune checkpoints, and macrophage polarization and found that FZD2 or FZD6 expression was strongly and positively correlated with immune checkpoints. TAMs are widely accepted as macrophages clustered in the TME and promote tumor progression (Chen et al., 2015). It is well known that TAMs can be polarized into the M1-type (classically activated) and the M2-type (alternatively activated immune-suppressive type). Previous studies demonstrated that infiltration of TAMs in gliomas is mainly determined by the M2-type (Badie and Schartner, 2000). The significance of TAMs in cancer development has also been proven in other studies, in which high infiltration of TAMs in most tumor types, including breast cancer, gastric cancer, lung cancer, hepatoma, and other malignancies, was associated with a poor prognosis (Cardoso et al., 2014; Zhao et al., 2017; Wang et al., 2018). In the current study, the expressions of FZD2 and FZD6 (especially FZD2) were strongly and positively correlated with M2-type macrophage polarization, and lead to an immunosuppressive phenotype. DCs are crucial for boosting protective immunity since they are known to trigger pathogen-specific T-cell responses. DCs identify, collect, and deliver antigens to T-cell throughout the adaptive immune response process. They also upregulate costimulatory molecules and create inflammatory cytokines. They eventually migrate to secondary lymphoid organs to present the antigen to T-cell. Similar to TAMs, DCs may be divided into distinct subtypes. Our findings verified that Th2 and Th17 infiltration led to the immune-suppressive subtypes of DCs (Collin and Bigley, 2018). In addition, neutrophil infiltration was noted. Up to 70% of circulating leukocytes are neutrophils, which serve as the first line of defense against infections (Wang et al., 2014). The type of tumor and the stage of tumor development affect the neutrophil phenotype in the setting of cancer. They exhibit an immunosuppressive character as the tumor grows. Together, FZD2 and FZD6 demonstrated a close relationship with immune cell infiltration, which warrants further investigation.

## 5 Conclusion

In summary, our preliminary findings revealed that FZD1/2/5/6/7/8 may be a disadvantageous factor for glioma, whereas FZD2/6 may serve as a novel independent predictor of poor prognosis.

## Data Availability

The datasets presented in this study can be found in online repositories. The names of the repository/repositories and accession number (s) can be found in the article/Supplementary Material.
